# A mini case report: *Klebsiella pneumoniae*-induced metastatic neck abscess following laparoscopic appendectomy

**DOI:** 10.1016/j.heliyon.2024.e31062

**Published:** 2024-05-10

**Authors:** Cancan Jin, Jiangnan Hu, Linshu Wang, Sizhe Hu, Kangyi Wang, Liangbin Fu, Xiaokang Zhao, Feng Qian, Hui Shentu

**Affiliations:** a Department of Oncology, Affiliated Dongyang Hospital of Wenzhou Medical University, Dongyang, 322100, China; b Department of Surgery and Stanford Cancer Institute, Stanford University, Stanford, CA, 94305, USA; c Department of NeuroSurgery, Stanford University, Stanford, CA, 94305, USA; d Department of Oncology, Affiliated Dongyang Hospital of Wenzhou Medical University, Dongyang, 322100, China

**Keywords:** Laparoscopic appendectomy, *Klebsiella pneumoniae*, Metastatic abscess

## Abstract

Common complications following laparoscopic appendectomy include wound infection, bleeding, intra-abdominal abscess, small bowel obstruction, stump leakage, and stump appendicitis. Here, we presented a case reporting detailing a rare complication following laparoscopic appendectomy: the development of a metastatic neck abscess induced by *Klebsiella pneumoniae(K. pneumoniae)*. A 49-year-old male underwent emergency laparoscopic surgery with prophylactic antibiotic administration for acute appendicitis. Subsequently, he experienced persistent neck pain and fever postoperatively, prompting further investigation. Pus and blood cultures revealed *K. pneumoniae*, with magnetic resonance imaging confirming the presence of a neck abscess. Antibiotic therapy was adjusted, and surgical drainage of the abscess was performed after multidisciplinary consultation. The patient was discharged without complications. While rare, metastatic abscesses following appendectomy warrant consideration, particularly in *K. pneumoniae* infections. Comprehensive clinical assessment, imaging, and laboratory evaluation are crucial for timely diagnosis and management of such complications.

## 1 Introduction

Appendicitis remains one of the most common abdominal diseases requiring surgical intervention; its yearly incidence is approximately 100 cases per 100,000 individuals worldwide, with an 8 % lifetime risk of disease [1]. It is readily diagnosed based on typical symptoms and physical examinations, combined with imaging and blood test results. Surgery in combination with antibiotics is the preferred treatment [2,3]. Gram-negative bacteria account for most of the microorganisms identified in pus cultures, and antibiotics are primarily used to treat them [4]. If the therapy is inadequate, complications may arise, such as bowel obstruction, stump leakage, intra-abdominal abscess, surgical site infection, and stump appendicitis [5]. Metastatic abscesses occurring secondary to acute appendicitis are rare, and neck metastasis is even more uncommon. Herein, we report such a case of a neck metastatic abscess after surgical appendectomy.

## 2 Case presentation

**Fig. 1:**
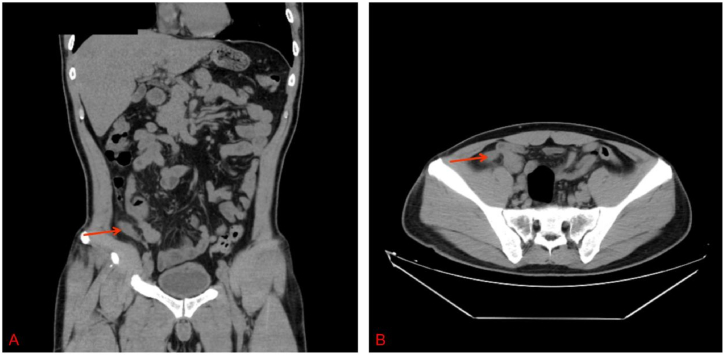
(A) A cross-sectional CT image revealing appendicitis. (B) A coronal section CT image showing appendicitis. CT, computed tomography.

**Fig. 2:**
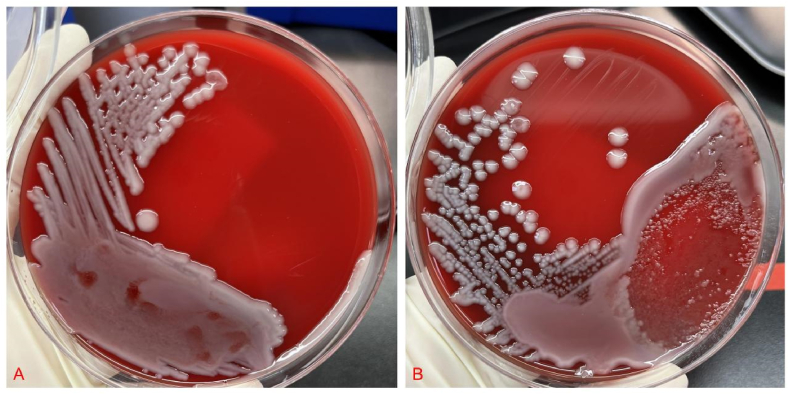
(A) Blood culture of *Klebsiella pneumoniae*. (B) Pus culture of *Klebsiella pneumoniae* from the appendix.

**Fig. 3:**
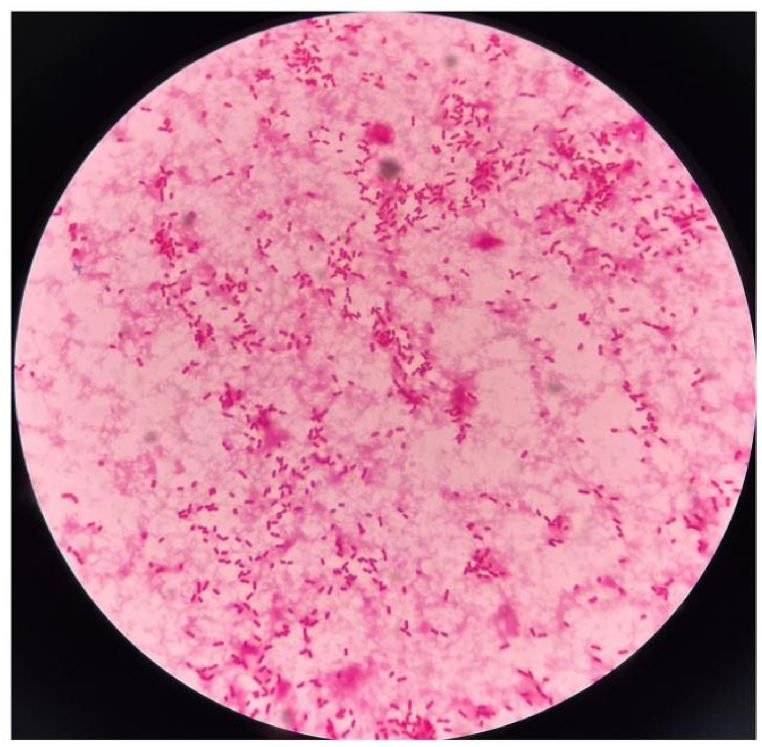
Image of Gram-stained *Klebsiella pneumoniae* from blood samples at 100 × magnification.

A 49-year-old man with lower quadrant pain that persisted for 65 h visited the emergency department. The patient's health had been good, and there had been no other medical visits. His vital signs were as follows: pulse, 116 beats/min; temperature 38.0 °C; blood pressure, 113/74 mmHg; and respiratory rate, 20 breaths/min. On physical examination, the patient presented with typical features of appendicitis, including right lower quadrant abdominal pain and rebound tenderness; no other abnormalities were found. Blood test results were as follows: leukocytes, 15.89 × 10^9^/L (leukocytosis); neutrophils, 80.4 %; C-reactive protein (CRP), 127.9 mg/dL; procalcitonin (PCT), 16.35 ng/mL; and other blood tests, normal. Plain abdominal computed tomography (CT) revealed thickened appendiceal wall layers and increased inflammatory exudate (Fig. 1(A and B)). Therefore, acute appendicitis was diagnosed, and laparoscopic appendectomy was successfully performed. Cefoperazone sodium/sulbactam sodium (2.0 g every 8 h) was prescribed as antibiotic treatment on admission. On the following day, blood and pus cultures from the appendix suggested the presence of *Klebsiella pneumoniae(K. pneumoniae)* exclusively, which was determined to be susceptible to cefoperazone sodium/sulbactam sodium by antimicrobial susceptibility testing (see Fig. 2, Fig. 3).

Unexpectedly, the patient then complained of neck pain, which gradually worsened over time. Fever was observed one day after surgery, with the body temperature reaching a maximum of 39.2 °C on the fourth day after surgery. Another round of tests was performed: leukocytes, 11.49 × 10^9^/L (leukocytosis); neutrophils, 69.6 %; CRP, 120.5 mg/dL; PCT, 2.14 ng/mL; and other tests, normal.

**Fig. 4:**
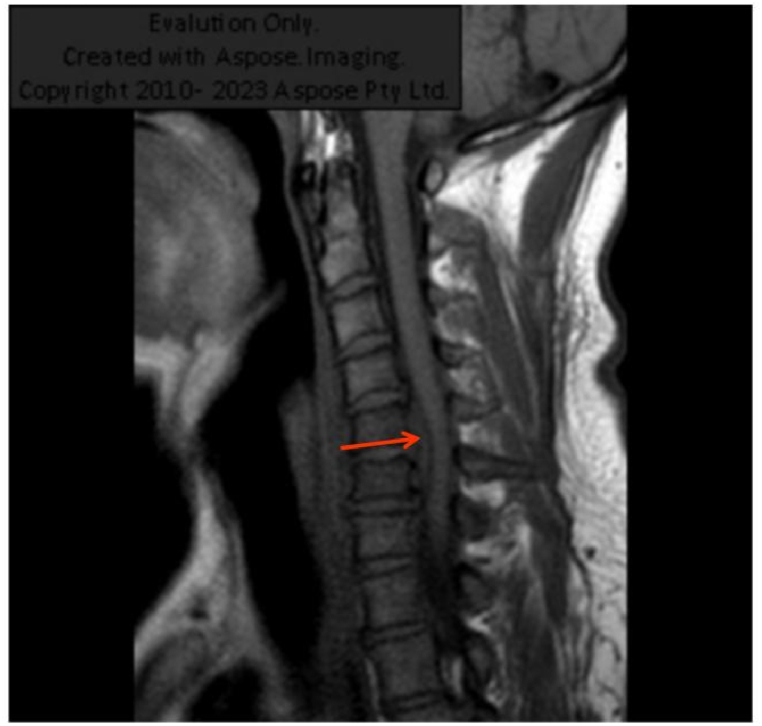
Neck magnetic resonance image showing an abscess.

**Fig. 5:**
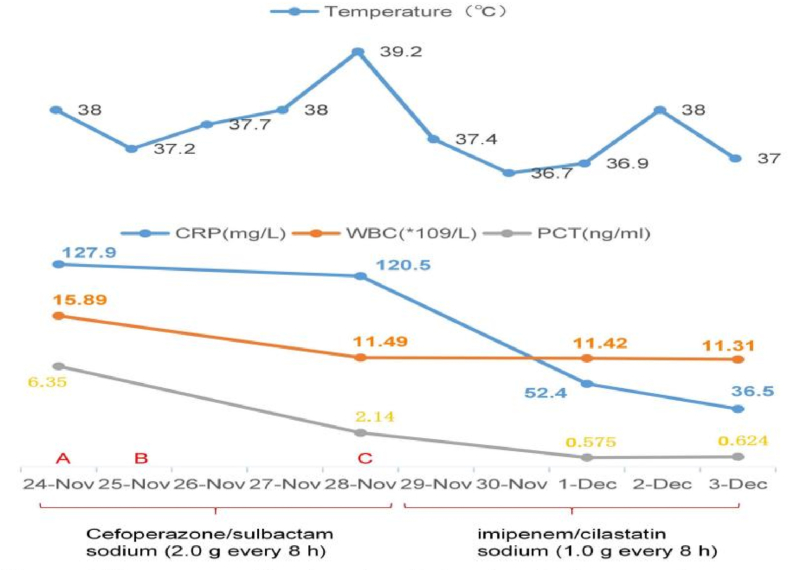
Time course of treatment and laboratory findings over time. (A) Surgery and cultures from appendix pus and blood were performed.(B) Patient began to complain about neck pain.(C) Blood cultures were performed again.

**Fig. 6:**
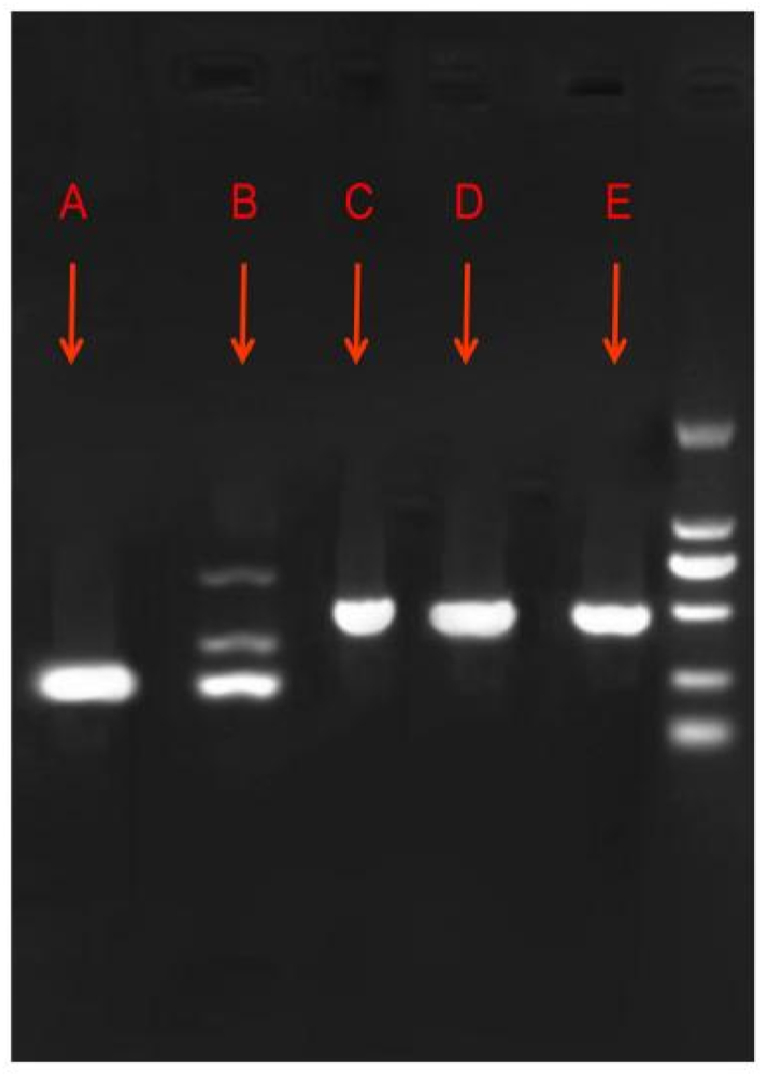
Gel electrophoresis images of different virulence genes of *Klebsiella pneumoniae* from the pus cultures. (A) *iroB* (235 bp), (B) *iucA* (239 bp), (C) *peg344* (508 bp), (D) *rmpA* (588 bp), and (E) *rmpA2* (430 bp). The PCR conditions and primers used have been described previously [6]. The expected band is identified in each panel with an arrow. The PCR results of the samples from blood and neck abscess are the same as the pus.

Considering the recurrent fever and high levels of inflammatory indicators, the antibacterial treatment was deemed ineffective. Thus, we switched the treatment from cefoperazone sodium/sulbactam sodium to imipenem/cilastatin sodium (1.0 g every 8 h) on the fifth postoperative day. Additionally, an abdominal CT was performed and showed no abnormalities. The patient had no other discomfort apart from the ongoing neck pain. A magnetic resonance imaging (MRI) scan was performed, and an abscess was found in the neck (Fig. 4). Therefore, we considered the diagnosis of *K. pneumoniae* bacteremia leading to metastatic abscess formation in the neck. After treatment, the inflammatory indicators decreased significantly; however, the neck pain did not improve by the seventh postoperative day. In light of this, a multidisciplinary team consultation was convened to review the infection's progression(Fig. 5(A,B,C)). Based on recommendations, infectious disease specialists and orthopedists were advised to perform surgical drainage of the abscess and manage the abscess tissue in the orthopedic ward. As expected, only *K. pneumoniae* was detected in the pus culture of the appendicitis site, neck abscess and blood samples, suggesting the likelihood of the same pathogen spreading throughout the system. Furthermore, we identified the virulence genes *iroB*, *iucA*, *peg344*, *rmpA*, and *rmpA2* in the isolated strains (Fig. 6), which a previous report has associated with hypervirulent bacterial phenotypes [6]. Despite having undergone two major surgeries, the patient was discharged without complications. At discharge, inflammatory markers were within normal limits, and the patient reported no neck pain. Given the absence of discomfort, imaging studies were not conducted further.

## 3 Discussion

The incidence of postoperative complications after appendicitis is generally 3.0–28.7 %, including bowel obstruction (0–1.9 %), surgical site infection (1.2–12.0 %), intra-abdominal abscess, stump leakage, and stump appendicitis [7]. Intra-abdominal abscesses after appendicitis are more common whereas metastatic abscesses are rarer. The patient most likely developed a metastatic abscess caused by *K. pneumoniae*, the most common opportunistic pathogen possessing both high pathogenicity and virulence. Classical *K. pneumoniae* mainly infects hospitalized and immunocompromised patients, while hypervirulent *K. pneumoniae* can infect young and healthy individuals and lead to disseminated infections. Through a comprehensive review, we identified comparable cases of metastatic infections induced by *K. pneumoniae* (Table 1). From these cases, we found that the most common site of dissemination was the liver, which may be identified as invasive liver abscess syndrome, commonly causing aggressive hepatic abscesses accompanied by symptoms such as endophthalmitis, necrotizing fasciitis, and meningitis [8,9]. Other common infections include lumbar or cervical spondylitis and discitis, lung abscesses, psoas muscle abscesses, splenic abscesses, neck abscesses, otitis media, osteomyelitis, arthritis, septic pulmonary emboli, and pylephlebitis [[10], [11], [12], [13], [14]]. However, there was no liver abscess in our case. Notably, our case did not involve a liver abscess. The primary objective of presenting this case was to highlight to clinicians that the disseminated infection of highly virulent *K. pneumoniae* can disseminate beyond the typical sites such as the eyes and brain. Therefore, when *K. pneumoniae* is identified in cultures, clinicians should consider the possibility of dissemination to other sites, such as neck metastatic abscess as reported in this case.

**Table 1:** Reports of multiple abscesses caused by *Klebsiella pneumoniae*.

No.	Sex/Age (years)	Infection Sites	Treatment	Outcome
1 [17]	Female/55	Blood, liver, and central nervous system	Antibiotics + drainage (liver)	Death
2 [18]	Female/64	Blood, liver, and eye	Antibiotics + drainage (liver)	Blindness in one eye
3 [10]	Male/78	Blood, liver, eye, and lumbar disk	Antibiotics + drainage (liver) + lumbar disk surgery	Blindness in one eye
4 [11]	Male/48	Blood, soft tissue, and eye	Antibiotics	Infection cleared
5 [12]	Male/71	Blood, liver, muscle, and prostate	Antibiotics	Infection cleared
6 [13]	Female/57	Blood, liver, lung, and central nervous system	Antibiotics + drainage (liver)	Death
7 [19]	Male/20	Blood, liver, and bilateral osteomyelitis	Antibiotics	Infection cleared
8 [20]	Female/69	Blood, liver, and spondylodiscitis	Antibiotics + hepatectomy + drainage (lumbar)	Infection cleared
9 [21]	Male/59	Blood, liver, left and right endophthalmitis, psoas abscess, and infectious spondylitis	Antibiotics + drainage (liver)	Infection cleared

Cultures of the preoperative blood, pus of the appendix, and pus of the neck abscess were all positive for *K. pneumoniae*, which indicated that the bacteria of the neck abscess originated from the appendix in this case. Neck abscesses rarely occur after surgical appendectomy. When the infection involves other sites, a specialist is required to manage such patients effectively. In this case, after discovering the neck abscess via MRI, infection specialists and an orthopedist were consulted before finally deciding to perform abscess drainage and removal. The presence of an abscess indicates that the targeted antibiotic treatment was ineffective before surgery. Therefore, surgical intervention is key to this treatment regimen. If not promptly recognized and treated, the abscess may be life-threatening or cause paralysis. If *K. pneumoniae* is detected in pus or blood, the clinical focus should include laboratory indicators, body temperature, and symptoms, including awareness of the possibility of metastatic abscess. Regrettably, we were not able to perform bacterial gene sequencing in our hospital, so we were unable to assess homology of the *K. pneumoniae* isolates. However, the bacteria from different cultures were only *K. pneumoniae* and had the same antibiotic susceptibility, suggesting the expression of similar genes (Table 2). The *rmpA*, *rmpA2*, *iroB*, *iucA*, and *peg344* genes have already been demonstrated to be expressed by hypervirulent *K. pneumoniae* strains [15], and they were identified in the *K. pneumoniae* strains found in the pus cultures from the appendix of our patient. Although simultaneous development of abscess at multiple sites may more commonly occurred in immunocompromised patients, it was also important to consider the possibilities. Considering this patient's immunocompetency, timing of symptom onset, and microbiological analysis, we believed the neck abscess was more likely resulted from metastasis.

**Table 2:** Antibiotic susceptibility profile of the *Klebsiella pneumoniae* from blood and pus samples (neck abscess and appendix).

Antibiotics	MIC (μg/mL)	Interpretation	Antibiotics	MIC (μg/mL)	Interpretation
Amikacin	≤2	S	Ertapenem	≤0.12	S
Amoxicillin/clavulanic acid	≤2	S	Cefuroxime	2	S
Cefepime	≤0.12	S	Imipenem	≤0.25	S
Cefoperazone/sulbactam	≤8	S	Levofloxacin	≤0.12	S
Cefoxitin	≤4	S	Piperacillin/tazobactam	≤4	S
Ceftazidime	≤0.12	S	Tigecycline	≤0.5	S
Ceftriaxone	≤0.25	S	Co-trimoxazole	≤20	S

The bacteria from cultures of blood and pus samples (neck abscess and appendix) were only *Klebsiella pneumoniae* and had the same antibiotic susceptibility.

MIC, minimum inhibitory concentration; S, susceptible.

The patient complained of neck pain on the first postoperative day, which did not attract significant attention. As the patient had no other discomfort apart from neck pain, it was first considered to be related to residual gas after laparoscopic surgery [16]. Typically, pain caused by the gas gradually decreases over time; however, the patient's symptoms gradually worsened. The pain was also accompanied by recurrent fever, with a body temperature that peaked at 39.2 °C on the fourth postoperative day. Additionally, the results of routine tests and CRP and PCT levels were abnormal; however, abdominal CT showed no abnormalities. While *K. pneumoniae* was known to cause a variety of infections, including pneumonia, urinary tract infections, and bloodstream infections, it was less common for it to cause abscesses specifically in the neck as the original site. Primary neck abscesses were more commonly associated with other pathogens such as *staphylococcus aureus* or *streptococcus species*. Therefore, we considered other reasons and found an abscess in the neck via MRI. Determining whether the abscesses developed separately or resulted from metastasis required a thorough clinical evaluation, including assessing the timing of symptom onset, imaging studies, and microbiological analysis. Additionally, molecular typing techniques such as whole-genome sequencing could help determine the relatedness of bacterial isolates from different sites, providing further insights into their origin and spread. This case underscored the importance of attentively addressing patient complaints in clinical practice, as they may signify underlying complications necessitating prompt intervention.

## 4 Conclusion

Metastatic abscesses after laparoscopic surgery are rare, which highlights the importance of timely and accurate diagnosis followed by appropriate treatment when dealing with *K. pneumoniae* infections to prevent treatment delays and alleviate patient discomfort. Even though metastatic abscesses are rare, the detection of *K. pneumoniae* in cultures should prompt consideration of potential disseminated infections. In conclusion, it is imperative not to overlook patient complaints to facilitate early diagnosis and intervention.

## Ethics statement

This study was reviewed and approved by Dongyang Hospital of Wenzhou Medical University, Dongyang, China (approval number:2023-YX-295].

The patient provided informed consent for the publication of the anonymized case details and images.

## Patient consent

Informed consent was obtained from the patient for the publication of the anonymized case details and images.

## Ethics declarations

This study was reviewed and approved by [Medical Ethics Committee of Dongyang People's Hospital], with the approval number: [Dongyang People's Hospital 2023-YX-295].

## Data availability statement

All data are included in the article/supplementary material and referenced in article.

## CRediT authorship contribution statement

**Cancan Jin:** Writing – review & editing, Writing – original draft, Visualization, Supervision, Resources, Conceptualization. **Jiangnan Hu:** Writing – review & editing. **Linshu Wang:** Writing – review & editing. **Sizhe Hu:** Writing – review & editing. **Kangyi Wang:** Writing – review & editing. **Liangbin Fu:** Writing – review & editing. **Xiaokang Zhao:** Writing – review & editing. **Feng Qian:** Writing – review & editing. **Hui Shentu:** Writing – review & editing.

## Declaration of competing interest

The authors declare that they have no known competing financial interests or personal relationships that could have appeared to influence the work reported in this paper.
